# Physiological roles of macrophages

**DOI:** 10.1007/s00424-017-1945-7

**Published:** 2017-02-09

**Authors:** Siamon Gordon, Luisa Martinez-Pomares

**Affiliations:** 1 Graduate Institute of Biomedical Sciences, College of Medicine, Chang Gung University, Taoyuan City, 33302 Taiwan; 2 Sir William Dunn School of Pathology, Oxford University, Oxford, UK; 3 School of Life Sciences, University of Nottingham, Nottingham, UK

**Keywords:** Macrophages, Homeostasis, Tissue heterogeneity, Physiology, Receptors, Phagocytosis

## Abstract

Macrophages are present in mammals from midgestation, contributing to physiologic homeostasis throughout life. Macrophages arise from yolk sac and foetal liver progenitors during embryonic development in the mouse and persist in different organs as heterogeneous, self-renewing tissue-resident populations. Bone marrow-derived blood monocytes are recruited after birth to replenish tissue-resident populations and to meet further demands during inflammation, infection and metabolic perturbations. Macrophages of mixed origin and different locations vary in replication and turnover, but are all active in mRNA and protein synthesis, fulfilling organ-specific and systemic trophic functions, in addition to host defence. In this review, we emphasise selected properties and non-immune functions of tissue macrophages which contribute to physiologic homeostasis.

## Introduction

“The constancy of the internal environment is the condition for full and independent life: the mechanism that makes it possible is that which assured that maintenance, within the internal environment, conditions necessary for the life of these elements” (Claude Bernard).

**Fig. 1:**
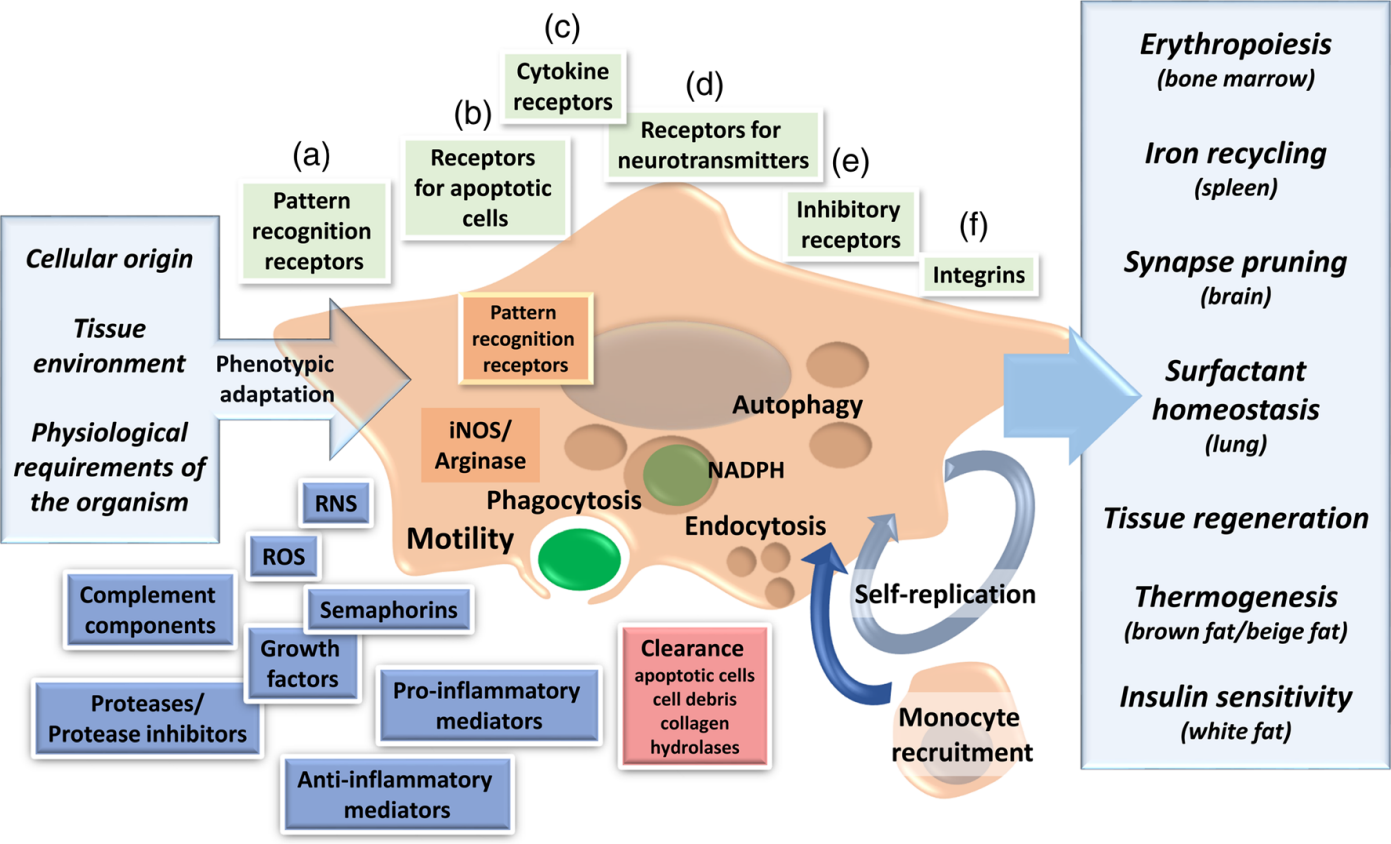
Physiological roles of macrophages and macrophages heterogeneity. Phenotypic heterogeneity of tissue macrophages is triggered by differences in cellular origin (self-replicated cells of embryonic origin vs monocyte-derived), tissue environment and adaptation to the physiological requirements of the organism (shown on the *left*). Macrophage heterogeneity underpins the vast range of physiological roles performed of these cells (shown on the *right*). Phenotypic heterogeneity is consolidated by tissue-specific “chromatin landscapes” in macrophages in response to environmental signals. Macrophages display a wide range of “sensor systems” in the form of *(a)* cell surface and intracellular pattern recognition receptors that detect microbe-associated molecular patterns and damage-associated molecular patterns; *(b)* receptors for apoptotic cells; *(c)* cytokine receptors such as IFN-γ and IL-4Rα that promote M1/M2-like activation, respectively; *(d)* receptors for neurotransmitters as well as *(e)* receptors that regulate macrophage activation in situ such as the inhibitory receptors SIRP1-α and CD200R. Integrins *(f)* mediate interaction with extracellular matrix. Macrophages produce numerous molecules that contribute to tissue remodelling and inflammation and mediate clearance of apoptotic bodies, cell debris and soluble compounds such as collagen and lysosomal hydrolases. High phagocytic and endocytic activities as well as motility are central to the ability of macrophages to support tissue-dependent functions. *RNS* reactive nitrogen species, *ROS* reactive oxygen species

The concept of homeostasis as a physiological principle, a term proposed by the neuroscientist Walter Cannon (1871–1945) and based on the work of Claude Bernard (1813–1878), has influenced the thinking of many macrophage investigators. Elie Metchnikoff (1845–1916), a comparative zoologist who initially was intrigued by intracellular digestion in invertebrates, became an experimental immunologist when he appreciated the role of phagocytes in tissue orthobiosis and inflammation [67]. Hans Selye (1907–1982) drew attention to the non-specific neuro-endocrine adaptations of the body to biological stress; however, the contributions of the mononuclear phagocyte system to the mechanisms of homeostasis and stress responses have not achieved full recognition. In this review, we consider topics related to the role of macrophages in development, metabolism and tissue-specific functions in different organs, to illustrate their remarkable ability to recognise and respond to the changing needs of the body. A general overview of the main topic covered is provided in Fig. 1.

Blood monocytes and tissue macrophages display many properties in common, but they adapt readily upon entering different tissue microenvironments, where they contribute to the general needs as well as specialised functions of individual organs. Reciprocal interactions between neighbouring cells and tissue macrophages are mediated by close contacts as well as secretory products which act at close range or distally via the circulation. Although primarily known as professional phagocytes [29], which engulf dying cells during development and subsequently during adult life, it has not been generally appreciated that macrophage activities extend beyond immunology and host defence. They express a broad repertoire of surface and intracellular receptors and produce a large variety of regulated bioactive molecules; together with their ability to enter all tissues, in the steady state and on demand, they constitute a dispersed organ system, able to influence and respond to every other system in the body.

## Cellular characteristics of the macrophage lineage: relevance to physiology

To set the stage for subsequent discussion, we briefly summarise some general properties of macrophages relevant to their functions in vivo. The cells originate from progenitors in the embryo and postnatally, as discussed further below, and are seeded throughout the body as relatively long lived, terminally differentiated haematopoietic cells. They are motile and display a range of membrane receptors to recognise and respond to a large repertoire of host-derived and foreign ligands [29]. These include adhesion molecules, opsonic and non-opsonic receptors to mediate phagocytic clearance of particulates, including micro-organisms and apoptotic cells, and for endocytosis of soluble ligands. In addition, they are able to sense and respond to the presence of microbial constituents, including RNA and DNA, within their cytoplasm, activating intracellular inflammasomes [32], caspase processing and release of IL-1β. A plethora of other cytokines, enzymes, growth factors, lipid-derived and low molecular metabolite products can also be secreted by macrophages in response to phagocytic and various stimuli and, in turn, act on macrophage receptors by autocrine mechanisms. Plasma membrane receptors co-operate in formation of a phagocytic synapse, excluding phosphatases such as CD45, and allow receptor kinases to initiate signalling pathways and cytoskeletal assembly, to bring about ingestion of particles [21]. A sequence of dynamic interactions with vesicular membranes derived from the endoplasmic reticulum and Golgi apparatus accompanies phagosome maturation, fusion with primary lysosomes, acidification and digestion [31, 39, 71]. Macrophages can internalise its entire surface membrane in 20 min, but recycle components equally rapidly to maintain membrane homeostasis [51]. Heterophagy refers to uptake of extracellular cargo; autophagy, the sequestration of effete cytoplasm and intracellular organelles, is well developed in macrophages [16]; genetic deficiency of lysosomal digestion or components of the autophagy pathway can result in storage diseases [59] or chronic granulomatous inflammation [68], respectively.

The uptake and killing of microbes and opsonised cellular targets by oxygen and nitrogen-derived radicals are mediated by assembly and activation of an NADPH oxidase and inducible nitric oxide synthetase, respectively [23, 43]. The ingestion of apoptotic targets, a major physiologic function of tissue-resident and inflammatory macrophages, is mediated by a range of redundant receptors, using exposed phosphatidyl serine and other cues as ligands [2]. Apoptotic cell uptake inhibits the inflammatory response associated with uptake of necrotic cells or microbes; anti-inflammatory products include TGF-β, IL-10 and PGE2 [38]. The importance of phagoptotic killing during phagocytosis has been emphasised by the studies of Guy Brown and colleagues [7]. Finally, macrophages can ingest membrane-enveloped nuclei or intracellular vesicles released from living cells or undergo cell fusion, to form multinucleated giant cells [35].

## Phenotypic adaptation of macrophages

Macrophage gene expression has been intensively studied in recent years and modules of genes regulated by a range of cytokines and other stimuli characterised in tissue-resident and inflammatory macrophages. Both pro- and anti-inflammatory pathways can be induced in macrophage populations, mediated by plasma membrane or cytoplasmic receptors. IFN-γ, for example, induces a characteristic profile of genes and proteins, often termed “classical or M1 activation” [47]; prototypic markers include upregulation of MHCII antigens, pro-inflammatory cytokines and induction of i-NOS, whereas CD206 expression is selectively downregulated. By contrast, interleukin-4 and interleukin-13, binding to a common IL-4 receptor alpha chain, induce an “alternative, M2” phenotype, characterised by upregulation of CD206, transglutaminase 2, arginase and, in the mouse, chitinase-like molecules such as YM1 [48]. An IL-13-specific receptor pathway has also been demonstrated by induction of macrophage homokaryon formation [62]. Phenotypic changes in macrophages are underpinned by metabolic reprogramming. M1 macrophages utilise Warburg metabolism and M2 macrophage oxidative metabolism [55]. The M1/M2 terminology arose as counterpart to Th1 and Th2 categories of adaptive immune responses, associated with the above and related cytokines. It is thought that instead of bimodal polarisation, a more complex phenotypic spectrum is more likely, especially in vivo [50, 52]. In truth, there is overlap with other cytokine-induced stimuli, e.g. after IL-10 stimulation or immune complex activation [19]. The stability or reversibility of phenotype in vivo needs a great deal of further study, at population and the single cell level. In addition, further studies on genetic and epigenetic mechanisms will contribute to a more sophisticated understanding of tissue-specific and generic macrophage phenotypic heterogeneity.

## Macrophage heterogeneity under physiological conditions

The immune system comprises a complex network of cellular and soluble components that collaborate to respond to challenges, both infectious and non-infectious, identified as potential threats to the organism. Accordingly, immune constituents are widely distributed throughout tissues with specialised lymphoid organs, such as splenic white pulp and lymph nodes, mostly providing unique sites for the induction of specific acquired immunity.

To date, newcomers to the field are still surprised to learn that healthy tissues incorporate immune cells, in particular, macrophages which are cells commonly associated with protection against infection and as mediators of tissue damage. Macrophages, unlike neutrophils, are integral components of tissues; they contribute to organ development and maintenance of homeostasis through mechanisms similar to those exploited during infection to detect pathogens and trigger inflammation. Widespread distribution of macrophages is accompanied by substantial tissue-specific phenotypic and gene expression profile differences related to the specific environmental cues in each organ that foster tissue-specific macrophage function [25]. Differences in gene expression are underpinned by the existence of distinct “chromatin landscapes” in each tissue macrophage population which are determined in large extent by the tissue environment [44]. Furthermore, fully differentiated macrophages may retain the capacity to change their gene expression profile when exposed to a new tissue environment [44], which highlights the potential ability of macrophages to undergo reprogramming even after differentiation. Phenotypically, there are instances of tissue adaptation in macrophages involving fine tuning of cellular activation thresholds. In mucosal tissues such as the respiratory and gastrointestinal tracts, macrophages display a higher threshold of activation as a means to minimise inflammation-mediated organ damage in response to repeated exposure to foreign material, including microbial products. In the case of alveolar macrophages, exposure to IL-10, surfactant proteins A and D and close interaction with respiratory epithelium through the inhibitory receptor CD200R and ανβ-6 tethered TGF-β, act as effective brakes of macrophage activation minimising the risk of unwanted inflammation [40]. In the gut, macrophages are hyporesponsive to stimulation [65]. Nevertheless, gut macrophages can trigger inflammation with one mechanism involving the detection of tissue damage through activation of the NLRC4 inflammasome which induces processing of pro-IL-1β constitutively expressed by intestinal macrophages [20]. This mechanism ensures detection of pathogens that cause tissue damage through the production of virulence factors [6, 70]. Macrophages in internal organs, less exposed to microbial constituents, present a lower activation threshold and are considered to be responsible for the induction of systemic inflammation in response to blood-borne pathogens, which can have deadly consequences in the case of sepsis [30].

## Origin of tissue macrophages

In addition to tissue-specific environmental cues, tissue-specific characteristics in macrophages can be linked in part to on site differentiation of macrophages from embryonic precursors during development and maintenance of macrophage numbers in tissues through self-renewal [26, 63] with imprinting of macrophage transcriptional programmes by tissue factors of particular importance [26]. Indeed, the original idea of monocytes as precursors of tissue macrophages that gave rise to the concept of “the mononuclear phagocyte system” proposed by van Furth et al. 1974 has been questioned by recent studies demonstrating that in many anatomical sites, tissue macrophages originate from precursors derived from the yolk sac during early embryogenesis with a second wave of embryonic precursors derived from foetal liver. This embryonic origin contrasts with the bone marrow origin for most immune cells [18, 26, 33]. Definitive haematopoiesis takes place in the bone marrow after birth. These findings were accompanied by the identification in the mouse of two populations of circulating monocytes: classical Ly6c^hi^ monocytes that originate directly from Ly6c + precursors and non-classical Ly6c^low^ monocytes that derive from Ly6c^high^ monocytes through the action of the transcription factor Nr4a1 [18]. Further, monocytes are now perceived to be not just transitional cells on their way to tissues; some authors refer to circulating Ly6C^lo^ monocytes as “blood macrophages” involved in the maintenance of the integrity of the endothelial barrier [33]. Ly6C^high^ monocytes contribute to replenishment of tissue macrophages with the rate of replenishment varying among anatomical sites. In the steady state, monocyte-derived tissue macrophages are almost non-existent in the case of brain as microglia are derived from yolk sac precursors. Similar scenarios have been observed in the case of Langerhans cells in the epidermis, alveolar macrophages in the lung and Kupffer cells in the liver. Monocyte-derived resident macrophages are more prevalent in the heart and pancreas (which display a slow replacement rate) and gut and dermis (which display a higher replacement rate) [26, 63].

Ly6C^high^ monocytes are recruited to tissues under steady state and inflammatory conditions. In the absence of inflammation, Ly6C^high^ monocytes sample the tissues and transport material to draining lymph nodes without differentiating into macrophages or dendritic cells [18]. Under inflammatory conditions, Ly6C^high^ monocytes differentiate into macrophages and can give rise to tissue macrophages with capacity for self-renewal. This process is highly dependent on the tissue-depletion strategy used and the organ under consideration [18].

## Role of macrophages in development

### Osteoclasts and bone remodelling

Bone remodelling occurs throughout life and is influenced by physical activity, diet and hormones. Bone generation, promoted by cells of mesenchymal origin termed osteoblasts, is balanced by the action of cells of haematopoietic origin termed osteoclasts that mediate bone reabsorption. Bone formation and degradation need to be balanced as disequilibrium would lead to osteoporosis (loss of bone mass) or osteopetrosis (excess bone mineralisation). Further, bone remodelling controls Ca^2+^ fluxes into and from the extracellular fluid which is required for the maintenance of the levels of calcium in blood [4]. Osteoclasts belong to the myeloid lineage, are M-CSF (CSF-1)-dependent and are multinucleated. Osteoclastogenesis is dependent on receptor activator of NFκB ligands (RANKL). Osteoclasts attach to the bone surface and assume a polarised morphology. The area attached to the bone is sealed, bone is degraded by the release of acid and acidic collagenolytic enzymes and released material is secreted at the opposite side of the cell through transcytosis [4]. Exchange of signals between osteoblasts and osteoclasts ensures that bone remodelling is regulated [64]. For instance, osteoblast apoptosis upon bone damage promotes osteoclast recruitment to the bone surface and osteoblast-derived RANKL is essential for osteoclast differentiation. IL-33 produced by osteoblasts inhibits osteoclast formation. Similarly, osteoclasts influence osteoblast function through the production of cardiotrophin-1 and semaphorin 4D (CD100), which promote and inhibit bone formation, respectively [64]. As a consequence of lack of bone resorption, bone marrow haematopoiesis does not occur due to lack of its anatomical niche. Csf1-deficient animals develop osteopetrosis and survive to adulthood because of extramedullary haematopoiesis in the spleen and liver and generation of osteoclasts takes place by compensatory expression of VEGF. Osteoclast generation enables the establishment of bone marrow haematopoiesis by enabling the creation of the haematopoietic niche [54].

Remodelling deficiencies in the absence of macrophages have been observed in mammary glands, kidney and pancreas, which suggest an important role in morphogenesis; this can be due to the contribution of macrophages to extracellular matrix remodelling and a role in maintenance of the viability and function of stem cells [73], as observed in mammary glands and liver.

### Macrophages and erythropoiesis

Definitive erythropoiesis occurs in the bone marrow; in particular, terminal erythropoiesis takes place in erythroblastic islands where tight clusters can be found between erythroid precursors with a central macrophage [8]. Molecules implicated in the interaction between erythroid precursors and macrophages include the erythroblast macrophage protein (Emp), the haemoglobin-haptoglobin receptor CD163, VLA-4 on erythroblasts and VCAM-1 on macrophages, and ICAM-4 on erythroblasts and αV integrin on macrophages. Erythroblastic islands have also been found in areas of definitive haematopoiesis such as foetal liver and splenic red pulp. Macrophages have been shown to promote erythropoiesis through the release of ferritin by exocytosis, which is internalised by erythroblasts where iron is released through acidification and proteolysis [15]; factors associated with chronic inflammation such as IFN-ɣ, TNF-α, TGF-β and IL-6 inhibit erythropoiesis [15]. Erythropoiesis is completely dependent on the phagocytic activity of macrophages. Macrophages phagocytose extruded erythrocyte nuclei which are released during the final phase of erythrocyte differentiation [15]. This is possible through the maintenance of adhesion molecules such as Emp and β1 integrin and exposure of phosphatidyl serine at the surface of the membrane-enveloped nuclei [15].

### Macrophages and brain development

Microglia constitute the resident macrophage population in the brain and spinal cord and are yolk sac-derived. In addition to responding to tissue injury, ramified microglia have been shown to contribute to synaptic maturation during brain development [58]. This process is termed “synaptic pruning” and involves the clearance of immature or defective neuronal synapses [1]; microglia dysfunction is now considered a major cause of neuropsychiatric or neurological disorders as exemplified by the link between mutations in the gene encoding CSF1R to hereditary diffuse leukoencephalopathy with spheroids [1]. Loss of function mutations in TREM-2, a receptor expressed by microglia in the CNS, have been associated with increased risk of developing late-onset Alzheimer’s disease [75]. Microglia protect against extension of amyloid fibrils by wrapping around amyloid-β plaques [11] and TREM-2 deficiency has been shown to disrupt this process [75].

### Macrophages and lung homeostasis

Alveoli are lined by the alveolar epithelium which is mainly composed of type I and type II alveolar epithelial cells. Type I epithelial cells cover 90% of the alveoli surface while type II epithelial cells are less abundant and mediate the synthesis, secretion and recycling of lung surfactant [5, 46]. Alveolar surfactant is composed of lipids and proteins and keeps alveoli open by reducing surface tension during breathing. Alveolar macrophages reside in the alveoli and establish close interactions with alveolar epithelial cells, some of which involve interactions such as CD200R-CD200 and CD47-SIPRα as inhibitory receptors. Surfactant proteins A and D also contribute to inhibition of cellular activation in alveolar macrophages by engaging SIRPα [37]. Alveolar macrophages are derived from embryonically derived foetal monocytes soon after birth through a process highly dependent on GM-CSF [18]. They replicate in situ and, thus, are independent from monocyte input in most instances [18]. Alveolar macrophages mediate 20–30% of surfactant lipid metabolism which explains the development of pulmonary alveolar proteinosis in GM-CSF-deficient mice and in mice lacking the ABC transporters (ABC-A1, ABCG1 or peroxisome proliferator-activating receptor gamma (PPAR-ɣ) which display inefficient surfactant catabolism in alveolar macrophages. These same genes are involved in cholesterol metabolism and transport by circulating monocytes and their deficiency has been linked to the development of atherosclerosis [46].

## Macrophages and metabolism

### Macrophages and metabolic syndrome

The involvement of macrophages in metabolic homeostasis was highlighted by major changes in macrophage numbers (10–15 to 45–60% of stromal cells) and phenotype (shift from alternative activation to classical activation) in white adipose tissue in obese insulin-resistant animals and humans [9, 17, 28, 49, 57]. These changes have also been observed in other metabolic tissues, liver and muscle, which implies a strong functional link between macrophage phenotype and parameters of tissue homeostasis [49]. This is in agreement with macrophages contributing actively to maintenance of tissue homeostasis, but also acting as sensors of tissue dysfunction and promoters of inflammation in the absence of infection. In the case of the metabolic syndrome, the central role of macrophages derives in part from the negative impact of inflammation on signalling events downstream of the insulin receptor, making the cells within metabolic tissues resistant to the action of insulin [49]. This effect in turn will lead to maladaptation in glucose metabolism, with increased insulin production by islet cells in the pancreas required to compensate for this inflammation-related effect and establishment of a pre-diabetic state [49]. In healthy white adipose tissue, insulin sensitivity in adipocytes is maintained through the action of resident macrophages that express the anti-inflammatory cytokine IL-10 acting as insulin sensitiser. These adipose-tissue macrophages have a phenotype consistent with alternative activation resulting from exposure to IL-4 and IL-13. PPAR-ɣ expression by macrophages is essential for control of alternative activation and improves insulin resistance [56]; eosinophils have been identified as the source of IL-4 in adipose tissue and lack of eosinophils has been associated with activation of adipose tissue macrophages and a tendency to develop insulin resistance in response to obesity [72]. Changes in macrophage phenotype in white adipose tissue after chronic increase in energy uptake occur in response to metabolic stress in the adipocytes; this leads to the recruitment of inflammatory monocytes by CCL2, CCL5 and CCL8 [73]. These inflammatory monocytes differentiate into classically activated macrophages, engulf dying adipocytes and further promote cellular activation. Cytokine production by these activated macrophages is considered major contributors to local and systemic insulin resistance. In the case of hepatic insulin resistance, Kupffer cells regulate the oxidation of fatty acids in hepatocytes; hepatic steatosis develops if Kupffer cells are unable to adopt an alternative activation phenotype because of loss of PPAR-δ in myeloid cells [73].

### Macrophages and iron recycling

Resident bone marrow macrophages and red pulp macrophages are essential for the process of erythrophagocytosis to recycle iron. Senescent or damaged erythrocytes are phagocytosed and digested to extract heme [22]. Upon uptake, heme is degraded through the action of heme oxygenase (HO; predominantly HO-1) to release iron into the cytoplasm. Cytoplasmic iron is exported by ferroportin located at the plasma membrane [22, 33]. Iron flux from macrophages exceeds iron adsorption through diet and iron stored in hepatocytes [22]. Intriguingly, heme itself has been shown to induce the development of F4/80^hi^, VCAM^hi^ iron recycling macrophages in bone marrow and spleen through induction of the transcription factor Spi-c [33]. Red pulp macrophages also express Nramp1 and the haptoglobin-haemoglobin receptor (CD163) which further emphasises their important role in systemic iron haemostasis [22]. There are several hypotheses regarding the signals recognised on aged erythrocytes by macrophages for their removal. These signals include the exposure of the universal apoptotic marker phosphatidyl serine in senescent erythrocytes, in spite of the fact that erythrocytes lack a cell nucleus and mitochondria and do not undergo canonical apoptotic process [15]. Other possibilities include the loss of CD47 expression, i.e. lack of an inhibitory signal, and conformational changes in CD47 that enable an interaction between CD47 and thrombospondin-1 which results in increased uptake by red pulp macrophages [15]. Splenic macrophages have also been implicated in the removal of intracellular inclusions in erythrocytes through a process that leaves the erythrocyte intact. These inclusion bodies are thought to be caused by oxidative damage [15].

## Macrophages and tissue regeneration

Self-contained inflammation with preservation of organ function depends on multifaceted interactions between macrophages and recruited immune cells, particularly neutrophils and monocytes that ensure timely resolution through changes in the activation state of macrophages (from a M1 to a M2-like phenotype). These constitute a “lipid mediator class switch” which shifts the production of lipid mediators from pro-inflammatory (for instance the eicosanoids prostaglandin E2, and leukotriene B4 that promote vasodilatation and chemotaxis) to pro-resolving lipids (such as lipoxins, resolvins, protectins and maresins), production of proteins such as annexin A1, gaseous mediators (carbon monoxide), chemokine inactivation and release of acetylcholine by the vagus nerve [34]. Resident macrophages dominate the induction of inflammatory responses in response to injury in the presence and absence of infection through activation of receptors by DAMPs and MAMPs. After stimulation, macrophages produce an array of cytokines such as TNF-α, IL-1β and IL-6 and chemokines as well as pro-inflammatory eicosanoids derived from arachidonic acid, an omega-6 fatty acid. Vasodilation and edema are followed by recruitment of neutrophils which are the first cells that enter the inflamed site. Neutrophil recruitment is favoured by the rapid exposure of P-selectin (CD62P) on activated endothelial cells followed by engagement of β2 integrins by the adhesion molecules ICAM-1 and ICAM-2, culminating in the process of diapedesis. Cues for directional cell migration are provided by the formation of chemokine gradients that guide the migrating neutrophil to the site of injury. Resolution of inflammation is now viewed as an active process and its de-regulation can hamper restoration of tissue function with lack of resolution leading to chronic inflammation and scar formation and fibrosis. The collaborative nature of the resolution process is illustrated by the requirement for transcellular metabolic events between leukocytes and epithelial cells and platelets for the synthesis of lipoxins, the first described pro-resolving lipids. Because of their potential for tissue damage, it appears counterintuitive to consider neutrophils’ essential partners for the resolution of inflammation. However, neutrophils promote resolution by enhancing monocyte recruitment by inducing changes in endothelial cells, for instance through shedding of IL-6Rα which complexes with IL-6. The IL-6Rα-IL-6 complex engages gp130 on endothelial cells and induces the production of E-selectin (CD62E) and VCAM1, thus promoting monocyte recruitment. In addition, apoptotic neutrophils change macrophage activation as uptake of apoptotic neutrophils is non-phlogistic, inducing IL-10, TGF-β and lipoxin synthesis by macrophages [66]. These changes are associated with the sensing of an “inflammation threshold” established early during the induction phase and highly dependent on the nature and extent of the original insult. Molecular mechanisms underpinning this concept include the fact that prostaglandin E2 induces changes in the lipoxygenase activity of neutrophils from 5-lipoxygenase to 15-lipoxygenase, required for synthesis of lipoxins [34]. During cardiac damage, inflammatory neutrophils and monocytes release oncostatin-M that induces the release by cardiomyocytes of Reg3β. Reg3β promotes the recruitment of reparative macrophages which favours the elimination of neutrophils [26]. In addition, macrophages control the neutrophil lifespan through the release of TNF-α, Fas ligand (FasL) and TRAIL [34].

The fate of the recruited monocytes and their contribution to repair depends on the organ and extent of damage. In the heart, recruited monocytes give rise to pro-inflammatory macrophages, while resident macrophages mediate tissue repair. In the spinal cord, both the pro-inflammatory and repair macrophages are monocyte-derived. While the pro-inflammatory macrophages derive from monocytes recruited through the CCR2-CCL2 pathway, repair-promoting macrophages derived from monocytes are recruited through a pathway involving VLA-4, VCAM-1 and CD73 [26]. In other scenarios, recruited monocytes have been shown to convert from an initial pro-inflammatory (M1) phenotype to a healing, pro-resolving (M2) phenotype [14, 60] consistent with plasticity of myeloid cells. This scenario has been observed in kidney [45] and muscle [3].

A key aspect during tissue regeneration and repair is the cross-talk between macrophages and stem cells. For instance, in liver, macrophage depletion has been shown to reduce invasiveness of liver progenitor cells into the parenchyma which is required for differentiation into hepatocytes [10]. In addition, macrophages can be induced to produce Wnt3a after uptake of hepatic debris. Wnt3a promotes expression of Numb in liver progenitor cells which inhibits Notch and promotes differentiation into hepatocytes instead of biliary cells [10]. Macrophages contribute to muscle regeneration with the sequential change from an early M1-like phenotype into a M2-like phenotype, essential for this process. Disruption of these changes in the phenotype of macrophages by blocking early inflammation (treatment with IL-10 or blocking IFN-ɣ) or late resolution (treatment with anti-IL-10) compromises muscle regeneration. Myogenic precursor cells are stimulated to migrate, grow and survive by macrophages with M1 cells stimulating proliferation and inhibiting fusion and M2 macrophages stimulating commitment of myogenic precursors and differentiation to form myotubes [10]. In kidney, macrophages have been found to produce Wnt7b which promotes repair of basement membrane [10].

Scar formation during wound healing can compromise organ function after injury and multiple reports have linked Th2 immunity to the regulation of tissue fibrosis, although the molecular processes of this effect are poorly understood [24, 74]. Knipper et al. [42] recently revealed an important cross-talk between macrophages and fibroblasts during wound healing in mouse injured skin, required to control the formation of collagen fibrils. This effect is dependent on the expression of IL-4-Rα by myeloid cells which, among others, induces the production of Relm-α, a well-characterised marker of macrophage activation in response to IL-4. IL-4Rα signalling during wound healing promotes the expression of Relm-α by macrophages, and Relm-α, in turn, induces the synthesis of the collagen-modifying enzyme lysyl hydroxylase 2 by fibroblasts, leading to fibrosis [42]. In line with the major influence of the tissue environment on macrophage biology, in liver and muscle, Th2 immunity promotes tissue regeneration through direct action on parenchymal cells [27, 36]; this suggests that macrophage contribution to wound healing is largely dependent on the intrinsic regenerative capacity of the tissue and, likely, the nature of the insult.

## Macrophages and thermogenesis

Brown fat, termed brown adipose tissue, generates heat (thermogenesis) and plays an important role in maintaining body temperature. In contrast to white adipocytes, brown adipocytes contain multilocular fat droplets, a large number of mitochondria, and express high levels of UCP1 (uncoupling protein 1). UCP1 is located in the inner membrane of the mitochondria and uncouples electron transport to promote heat instead of ATP in brown adipocytes. In humans, brown adipose tissue was thought to be restricted to neonates but recent data have shown the presence of brown fat in adults [12, 41, 69]. Adult human brown fat is dispersed in the supraclavicular, para-aortic and suprarenal regions, is cold-inducible and shares many characteristic with the cold-inducible beige fat found in the subcutaneous white adipose tissue of rodents [61]. Beige fat refers to the presence of white adipocytes functionally similar to brown adipocytes within white fat and promotion of beige fat formation is considered as a potential therapeutic tool in the fight against obesity [41]. Macrophages have been shown to be essential for adaptation to cold exposure that leads to lipolysis in white fat and thermogenesis in brown fat. The effect of macrophages depends on the acquisition of an alternative activated phenotype driven by lL-4/IL-13 and Stat-6 and that leads to secretion of catecholamines by these cells [53]. Indeed, IL-4 exposure of macrophages induces production of the three enzymes required for catecholamine production: tyrosine hydroxylase, dopa decarboxylase and dopamine β-hydroxylase [53]; the β3-adrenergic agonist CL-316246 was able to rescue the thermogenic defect in animals unable to produce IL-4 and IL-13 [53]. In agreement with these findings in mice, oral administration of the β3-adrenergic receptor agonist mirabegron can stimulate human brown fat thermogenesis in humans [13]. Macrophages, together with eosinophils and Th2 cytokines, have also been implicated in the remodelling of subcutaneous white fat into beige fat in mice [61].

## Conclusion and therapeutic opportunities

Macrophages are remarkable for their ability to sense their surroundings, produce a wide range of immune mediators alongside growth factors and opsonins, and their endocytic and phagocytic activity. The recognition of macrophages as intrinsic components of organs, adapted to the particular tissue environment and actively involved in homeostatic processes, opens the opportunity for macrophage-centred therapies that can be exploited to steer pathological processes towards outcomes that favour maintenance of organ function. Furthermore, the potential plasticity shown by these cells [44] makes it possible to envisage the possibility of using in vitro-differentiated cells to treat disease by adoptive transfer.
